# Cytotoxicity of combinations of the pan-KRAS SOS1 inhibitor BAY-293 against pancreatic cancer cell lines

**DOI:** 10.1007/s12672-022-00550-w

**Published:** 2022-09-01

**Authors:** Adelina Plangger, Barbara Rath, Sandra Stickler, Maximilian Hochmair, Clemens Lang, Lukas Weigl, Martin Funovics, Gerhard Hamilton

**Affiliations:** 1 Institute of Pharmacology, Medical University of Vienna, Vienna, Austria; 2 Karl Landsteiner Institute of Lung Research and Pulmonary Oncology, Klinik Floridsdorf, Vienna, Austria; 3 Department of Trauma Surgery, Sozialmedizinisches Zentrum Ost, Donauspital, Vienna, Austria; 4 Division of Special Anesthesia and Pain Medicine, Medical University of Vienna, Vienna, Austria; 5 Department of Cardiovascular and Interventional Radiology, Medical University of Vienna, Vienna, Austria

**Keywords:** Pancreatic cancer, KRAS, SOS1, BAY-293, Drug combinations

## Abstract

KRAS is mutated in approximately 25% of cancer patients and first KRAS G12C-specific inhibitors showed promising responses. Pancreatic cancer has the highest frequency of KRAS mutations but the prevailing KRAS G12D mutation is difficult to target. Inhibition of the GTP exchange factor (GEF) SOS1—KRAS interaction impairs oncogenic signaling independently of the specific KRAS mutations. In general, cell lines exhibiting KRAS mutations show specific alterations in respect to glucose utilization, signal transduction and stress survival. The aim of this investigation was to check the putative synergy of the SOS1 inhibitor BAY-293 with modulators targeting specific vulnerabilities of KRAS-mutated cell lines in vitro. The cytotoxicity of BAY-293 combinations was tested against MIA PaCa-2 (G12C), AsPC1 (G12D) and BxPC3 (KRAS wildtype) cell lines using MTT tests and calculation of the combination indices (CI) according to the Chou-Talalay method. The results show that BAY-293 synergizes with modulators of glucose utilization, inhibitors of the downstream MAPK pathway and several chemotherapeutics in dependence of the specific KRAS status of the cell lines. In particular, divergent responses for BAY-293 combinations between pancreatic and NSCLC cell lines were observed for linsitinib, superior inhibitory effects of trametinib and PD98059 in NSCLC, and lack of activity with doxorubicin in case of the pancreatic cell lines. Phosphoproteome analysis revealed inhibition of distinct signaling pathways by BAY-293 for MIA PaCa-2 on the one hand and for Aspc1 and BH1362 on the other hand. In conclusion, BAY-293 exhibits synergy with drugs in dependence of the tumor type and specific KRAS mutation.

## Introduction

Around 85% of pancreatic cancers are pancreatic ductal adenocarcinomas (PDACs) [1]. PDAC is one of the leading and increasing causes of cancer-related death, with less than 10% of patients surviving 5 years after diagnosis [2, 3]. Poor outcomes are due to late detection, metastatic spread, high recurrence rate and resistance to chemotherapy [4]. Mutant KRAS is essential for PDAC tumorigenesis and growth establishing PDAC as the most RAS-addicted cancer [5, 6]. Treatment for PDAC consist of administration of conventional cytotoxic drugs in absence of effective targeted therapy so far [7, 8]. Standard of care for PDAC has been gemcitabine, followed by nab-paclitaxel-gemcitabine combination and the extremely toxic FOLFIRINOX (folinic acid, fluorouracil, irinotecan and oxaliplatin) regimen [9]. Whereas surgical resection may have a curative effect in early stages, almost 80% of those undergoing surgery succumb to disease recurrence.

The most frequent genetic alterations in PDAC are activating mutations in *KRAS* and inactivating mutations in *CDKN2A*, *TP53*, and *SMAD4* with an incidence of approximately 85%, > 50%, 60–70%, and 50%, respectively [10]. Genes affecting epigenetic regulation and the DNA damage response are mutated at lower frequencies [11, 12]. These recurrently mutated genes alter several signaling cascades involving KRAS, G1-S cell cycle checkpoint, TGF-β, and WNT [13]. The most frequent driver mutations in *KRAS* are G12D and G12V accounting for 80–90% of KRAS alterations in PDAC [14–16]. Thus, successful targeting KRAS G12C in NSCLC employing sotorasib (AMG 510) and adagrasib (MRTX849) has a minor impact for PDAC that exhibit G12C mutations in 1–2% of cases [17–19]. However, Mirati Therapeutics showed partial responses in 5 patients and a disease control rate of 100% in 10 *KRAS* G12C mutant pancreatic cancer patients [20].

Non-*KRAS* G12C mutations may be targeted by other inhibitors or in an indirect manner instead of covalent binding of the G12C inhibitors to the cysteine residue of the active center. RAS proteins exchange GDP for GTP for activation and the GEF SOS1 activates KRAS by increasing this GTP turnover [21, 22]. Thus, the activity of SOS1 regulates the fraction of active state KRAS and proliferation [23, 24]. Several small molecule inhibitors that impair the interaction between SOS1 and KRAS have been developed [25, 26]. In various KRAS-mutant cells, SOS1 inhibitors result in high reduction of p-ERK activity and in KRAS wildtype cells, the Ras/MAP kinase pathway may be blocked completely. Therefore, SOS1 inhibitors have to be checked for a potential widespread toxicity in normal cells [21, 27]. However, GEF modulators are attractive for their pan-RAS inhibition independently of the type of KRAS mutations [28]*.* Besides non-G12C KRAS mutations, amplifications of native KRAS are frequently found in pancreatic cancer.

BI-3406 is a potent SOS1 inhibitor that is orally bioavailable and active in cells with KRAS mutations, but not on KRAS wild-type cells [21]. BAY-293 is a SOS1 inhibitor with high affinity but moderate antiproliferative activity in vitro that showed synergistic effects with the KRAS G12C inhibitor ARS853 [25]. Inhibition of SOS1 can increase the chemosensitivity of *KRAS*-amplified cancer cells to MEK inhibition and the SOS1 inhibitor BI-3406 has been demonstrated to synergize with the MEK inhibitor trametinib [21, 29]. Among the small molecule SOS1 inhibitors, only BI-1701963 has progressed into a phase I trial, either alone or combined with trametinib for KRAS-mutated tumors (NCT04111458) [30–32].

Targeting of KRAS in pancreatic cancer except for the G12C mutation may be achieved by using SOS1 inhibitors that as single drug are expected to possess limited anticancer activity. Therefore, combinations with compounds that hit specific vulnerabilities of KRAS-driven cells may provide sufficient clinical efficacy for significant tumor treatment. For the present investigation, the SOS1 inhibitor BAY-293 was used to check for synergism with a range of compounds targeting signaling pathways, metabolism and DNA damage. BAY-293 is not optimized for clinical use but can be employed as test compound that shares a similar chemical core structure with the BI-3406 SOS1 inhibitor. The results obtained for the G12C-mutated MIA PaCa2 and the G12D-mutated pancreatic cancer cell lines are compared to the BxPC3 cell line that exhibits an unusual *KRAS* wildtype [33]. Promising BAY-293 drug combinations have been previously published by our group for NSCLC cell lines but may be different in a pancreatic cancer cell background [34]. Furthermore, while our studies are performed with cells in 2D-culture, 3D NSCLC spheroids have been reported to exhibit markedly increased effects in response to EGFR-TKIs in combination with deletion of SOS1 or SOS1 inhibition by BAY-293 [35].

## Materials and methods [34]

### Chemicals

Chemicals were obtained from Sigma-Aldrich (St. Louis, MO, USA) or from Selleck Chemicals (Houston, TX, USA). Dulbecco’s phosphate buffered saline (PBS) was purchased from Gibco/Invitrogen (Carlsbad, CA, USA). Compounds were prepared as stock solutions of 2 mg/mL in either DMSO or 0.9% NaCl for cisplatin and aliquots stored at − 20 °C. Equivalent concentrations of DMSO were supplemented for medium controls.

### Cell culture

Permanent cell lines BxPC3, MIA PaCa-2 and AsPC-1 were obtained from the American Type Culture Collection (Rockville, MD, USA). The BH1362 KRAS G12C NSCLC cell line was established from a pleural effusion according to the Ethics Committee EK-21-210-1221 of the Viennese Hospital Association. Aforesaid cell lines were cultured in RPMI-1640 medium supplemented with 10% FBS (Biochrome, Berlin, Germany) and Penicillin/Streptomycin (Sigma-Aldrich). Upon confluence cells were detached with trypsin/EDTA (Sigma-Aldrich) and counted with a LUNA cell counter (Biozym, Vienna, Austria).

### Cytotoxicity assay

Aliquots of 1 × 10^4^ cells in 200 µL medium were treated for four days with twofold dilutions of the test compounds in 96-well microtiter plates in quadruplicate (TTP, Trasadingen, Switzerland). The plates were incubated under tissue culture conditions and cell viability was measured using a modified MTT (3-(4,5-dimethylthiazol-2-yl)-2,5-diphenyltetrazolium bromide) assay (EZ4U, Biomedica, Vienna, Austria). Optical density was measured using a microplate reader at 450 nm and values obtained from control wells containing cells and media alone were set to 100% proliferation. IC50 values were calculated using the Origin software (Originlab, Northampton, MA, USA). For the assessment of the interaction of the test compounds, tests were performed comprising the individual drugs alone and in combination, followed by analysis using the Chou-Talalay method with help of the Compusyn software (Compusyn Inc., Paramus, NJ, USA). The combination index CI < 0.9 indicates synergism, CI > 1.1 indicates antagonism and 0.9 < CI < 1.1 indicates an additive effect.

### Western blot array

The phosphorylation status of the cancer cell proteins was assessed using the Proteome Profiler Human Phospho-Kinase Array Kit (R&D, Minneapolis, MN, USA) recognizing 37 kinase phosphorylation sites and two related proteins. The kit was performed according to the manufacturer´s instructions and spots evaluated using the ImageJ and Origin software.

### Statistics

Statistical analysis was performed using Student’s t test for normally distributed samples (p < 0.05 was regarded as statistically significant). Values are shown as mean ± standard deviation (SD). Statistical significance is marked by an asterisk (*).

## Results

### Cytotoxicity of BAY-293

**Fig. 1:**
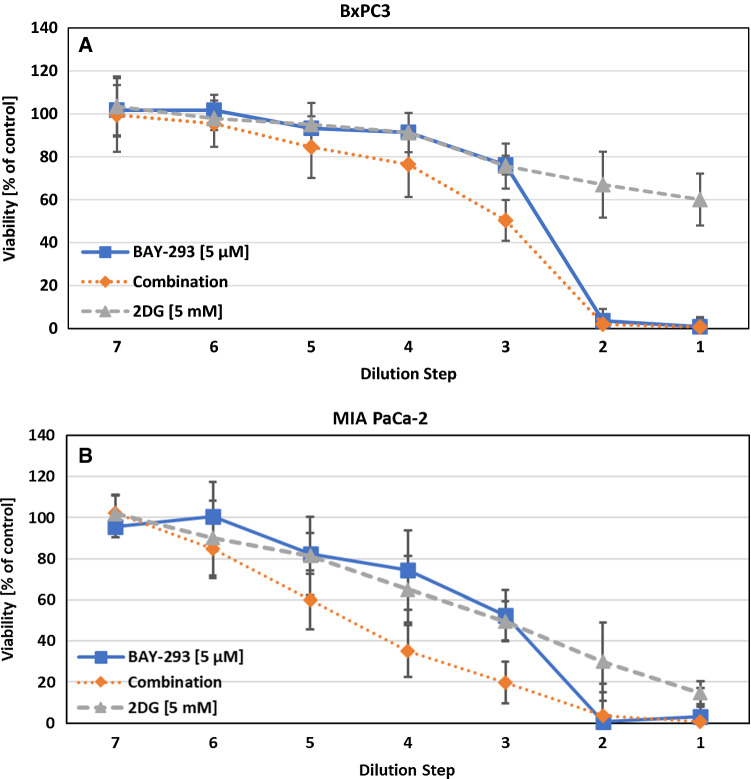
**A** Combination of BAY-293 with the glucose inhibitor 2-DG tested against BxPC3 cells. The initial concentrations of the compounds were diluted twofold in 7 steps. Values represent mean values ± SD. The CI-value for this combination was 0.670 ± 0.151. **B** Combination of BAY-293 with 2-DG tested against MIA PaCa-2 cells. Values represent mean values ± SD. The CI value for this combination was 0.697 ± 0.109

The activity of BAY-293 was assessed on all cell lines, namely BxPC3, MIA PaCa-2 and AsPC-1. The IC_50_ was 2.07 ± 0.62 µM for BxPC3, 2.90 ± 0.76 µM for MIA PaCa2 and 3.16 ± 0.78 µM for AsPC-1. BxPC3 is wildtype for KRAS while the other two cell lines have a *KRAS* G12C and a G12D mutation, respectively. After determination of the cytotoxic effects of BAY-293 a possible synergistic or antagonistic effect was tested using a range of compounds. Figure 1 shows the dose response curves of BAY-293 and 2-deoxyglucose (2-DG) for the cell lines BxPC3 and MIA PaCa-2, respectively. Initial concentrations were 5 µM for BAY-293 and 5 mM for 2-DG and these test compounds were diluted in 7 twofold steps. This combination yielded synergistic effects in both cell lines.

**Fig. 2:**
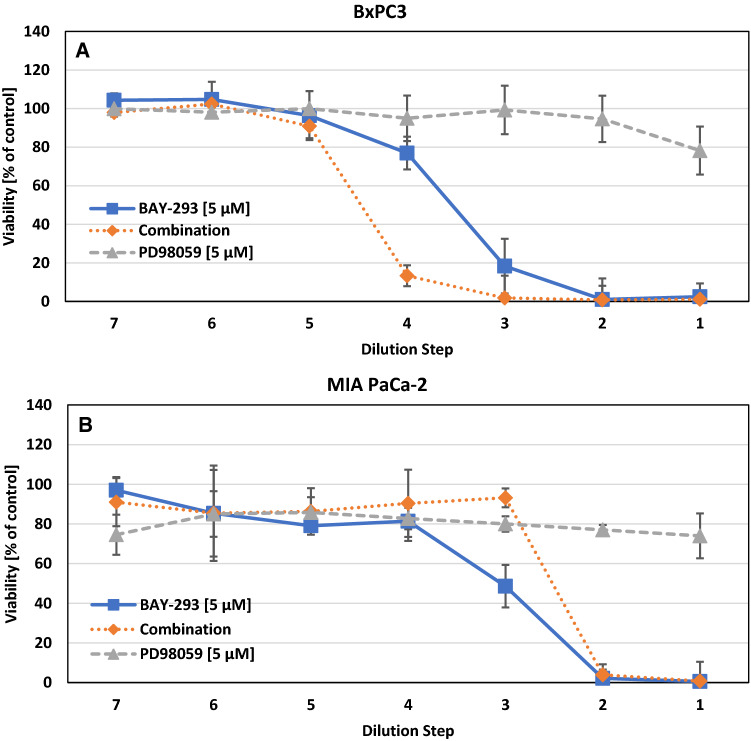
**A** Combination of BAY-293 with PD98059 against BxPC3 cells. The initial concentrations of the test compounds were diluted twofold in 7 steps. Values represent mean values ± SD. The CI-value was 0.564 ± 0.165. **B** Combination of BAY-293 with MEK kinase inhibitor PD98059 against MIA PaCa-2. Values represent mean values ± SD. The CI-value was 1.084 ± 0.032

In Fig. 2 the dose response curves for the combinations of BAY-293 and PD98059 (PD059, a MEK inhibitor), for BxPC3 and MIA PaCa-2 are shown. This combination yielded a synergistic effect for BxPC3 for the dilution steps 3 and 4. The CI value for this combination was 0.564 ± 0.165. In contrast, this combination proved to be antagonistic for MIA PaCa-2 with a CI value of 1.084 ± 0.032.

### Combination indices for BAY-293 drug combinations

**Fig. 3:**
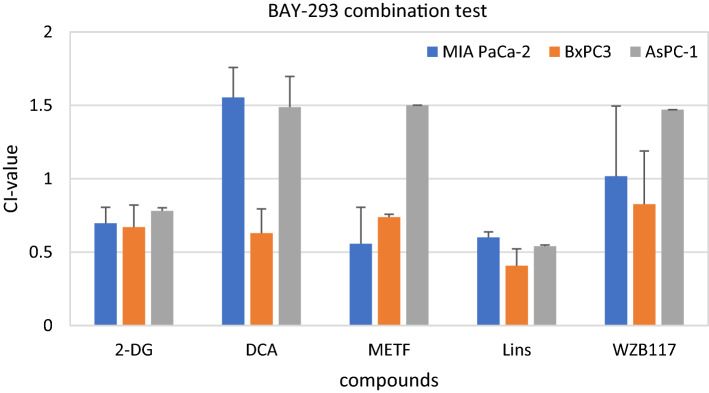
Cytotoxicity of combinations of BAY-293 with modulators of glucose utilization. Values present mean values ± SD. All tested combinations with these compounds showed statistically significant synergistic or antagonistic effects, with the exception of the combinations of WZB117 against MIA PaCa-2 and BxPC3, in relation to CI = 1 (1.08 ± 0.09)

The CI values were calculated for all three cell lines and 14 different modulators. We grouped the modulators according to their targeted pathway. BAY-293 was tested for antiproliferative effects in combination with 2-DG, dichloroacetate (DCA), metformin (MET), linsitinib (Lins) that modulate glucose utilization and the signaling of insulin/insulin-like receptors as well as WZB117, a glucose transporter 1 (GLUT1) inhibitor, respectively. Initial concentrations were 5 mM for 2-DG, 10 mM for DCA, 5 mM for metformin, 5 µM for linsitinib and 2 µM for WZB117. In Fig. 3 CI values for modulators related to glucose utilization are shown. Combining BAY-293 with 2-DG and linsitinib yielded synergistic antitumor activity in all three cell lines, respectively. DCA was only active in BxPC3, the effect of this combination was highly antagonistic in MIA PaCa-2 and AsPC-1 cells. Metformin (METF) exhibited synergy in MIA PaCa-2 and BxPC3 cell lines while showing antagonistic effects in AsPC-1. The combination of WZB117 with BAY-293 showed antagonistic effects in AsPC-1, additive effects in BxPC3 and MIA PaCa-2 cells.

**Fig. 4:**
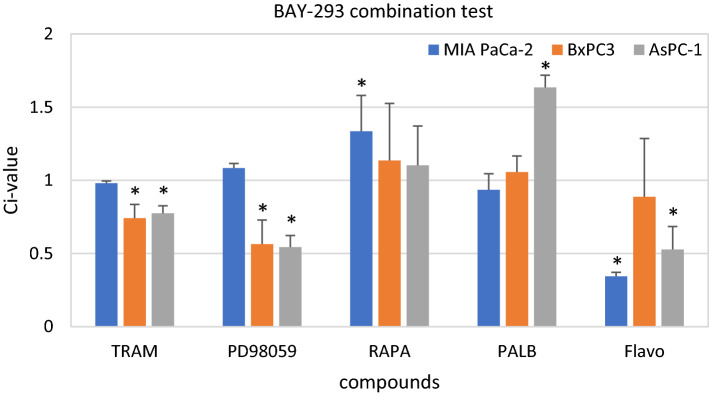
Cytotoxicity of combination of BAY-293 with MEK inhibitors trametinib and PD98059, mTOR inhibitor rapamycin and CDK inhibitors palbociclib and flavopiridol. Values present mean values ± SD. Statistically significant differences from CI = 1 (1.08 ± 0.09) are marked by an asterisk

The next group of compounds tested were the MEK inhibitors, namely trametinib (TRAM) and PD98059, the mTOR inhibitor rapamycin (RAPA), the CDK4/6 inhibitor palbociclib (PALB) and the pan-CDK inhibitor flavopiridol (Flavo). Initial concentrations were 50 µg/ml trametinib, 5 µM PD98059, 5 µM rapamycin, 10 µM palbociclib and 1 µM flavopiridol. TRAM and PD98059 exhibited a synergistic effect in BxPC3 and AsPC-1 and an additive effect in MIA PaCa-2 when combined with BAY-293, respectively (Fig. 4.). BAY-293 combined with RAPA yielded an antagonistic effect in MIA PaCa-2 and an additive effect in the other two cell lines. PALB revealed an additive effect in MIA PaCa-2 and BxPC3 and a strong antagonistic effect in AsPC-1 cells. The combination of Flavo showed synergism in MIA PaCa-2 and AsPC-1, but only an additive effect in BxPC3.

**Fig. 5:**
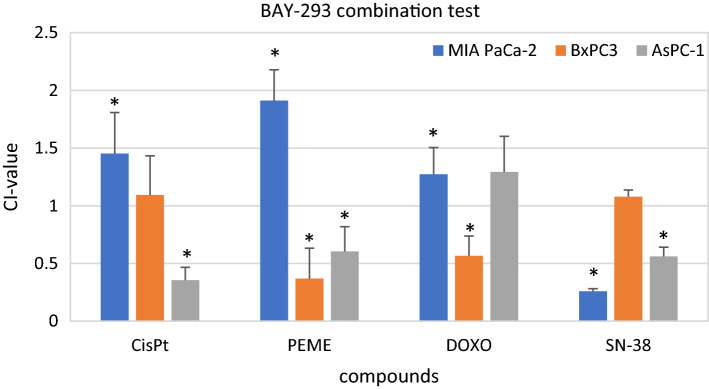
Cytotoxicity of combination of BAY-293 with cisplatin (CisPt), pemetrexed (PEME), doxorubicin (DOXO) and SN-38. Values present mean values ± SD. Statistically significant differences from CI = 1 (1.08 ± 0.09) are marked by an asterisk

The last four compounds tested are cisplatin (CisPt), pemetrexed (PEME), doxorubicin (DOXO) and SN-38 (see Fig. 5). The former three are chemotherapeutic drugs, the latter is a DNA topoisomerase 1 inhibitor. The initial concentrations were 10 µg/ml for cisplatin, 62,5 µg/ml for pemetrexed, 2 µg/ml for doxorubicin and 2.5 µM for SN-38. BAY-293 combined with cisplatin only yielded a synergistic effect in AsPC-1. The other two cell lines showed an additive and antagonistic effect, respectively. PEME revealed antagonism in MIA PaCa-2. However, BxPC3 and AsPC-1 yielded a synergistic effect with the combination of BAY-293 and PEME. DOXO revealed synergism in BxPC3 and SN-38 showed synergism in MIA PaCa-2 and AsPC-1.

### Effects of BAY-293 on kinase protein phosphorylation

**Fig. 6:**
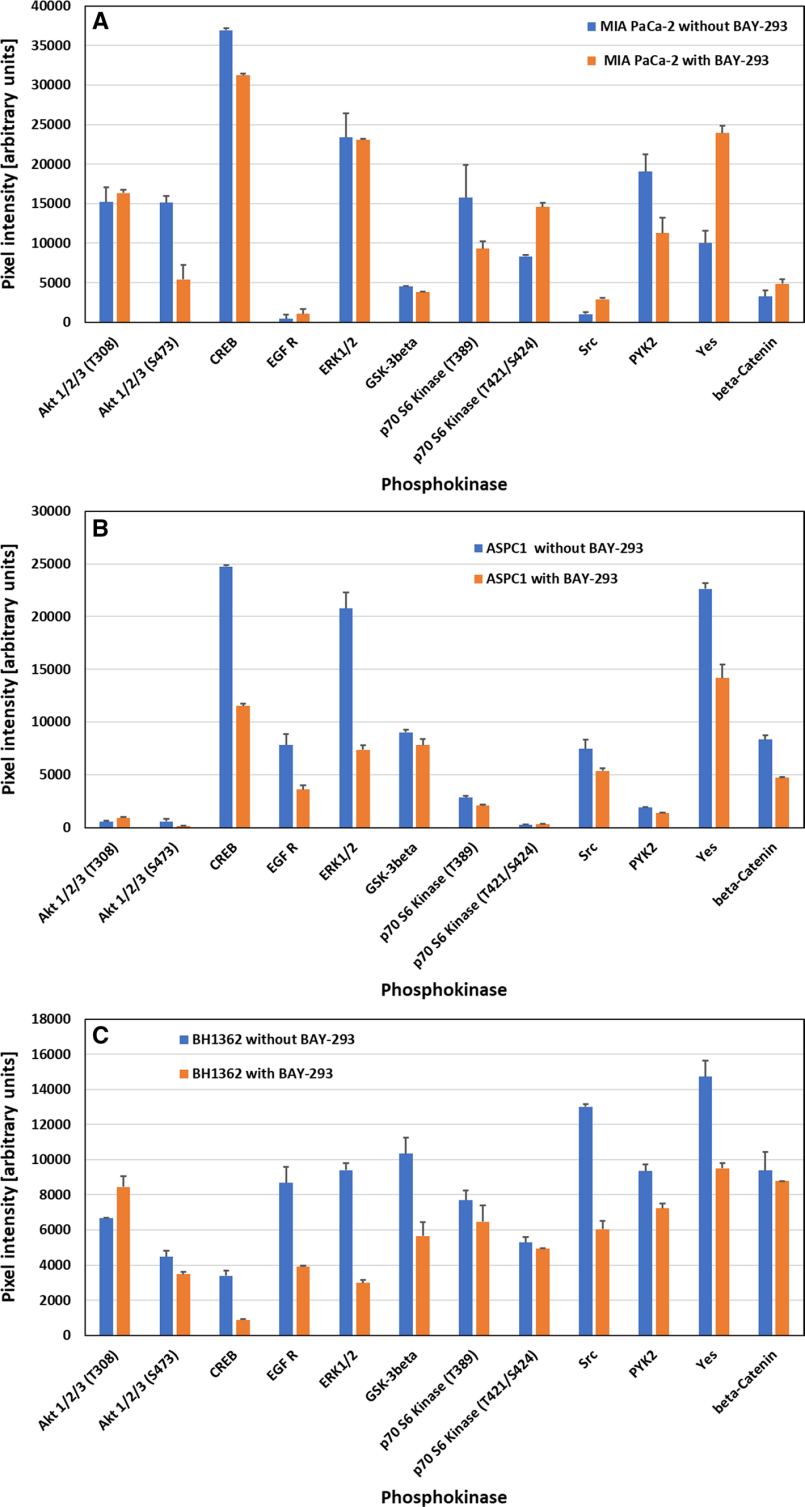
**A**–**C** Changes in phosphorylation of selected kinases in MIA PaCa-2 (**A**), Aspc1 (**B**) and BH1362 (**C**) cells in response to BAY-293 (2 µM). Data are show as mean values ± SEM. In case of MIA PaCa-2 all values are significantly different except for AKT 1/2/3 (T308) and ERK1/2. For Aspc1 all values are significantly different except for AKT 1/2/3. For BH1362 all differences are significant except for P70 S6 kinase and beta-catenin

The phosphorylation of selected kinases of MIA PaCa-2 and Aspc1 pancreatic cancer cell lines were compared to BH1362 NSCLC cell line carrying a KRAS G12C mutation for controls and cells pretreated with BAY-293 in Western Blot arrays. In MIA PaCa-2 cells Akt shows decreased phosphorylation at S473 (unchanged at T308) and CREB exhibited a minor decrease in phosphorylation (Fig. 6A). P70 S6 kinase revealed hypophosphorylation at T389 and increased phosphorylation at T421/S424. Src kinases Src and Yes are activated, whereas the PYK2 kinase showed decreased phosphorylation.

In Aspc1 cells treatment with BAY-293 resulted in marked hypophosphorylation of CREB, EGFR and ERK1/2, whereas AKT showed low phosphorylation (Fig. 6B). Likewise, the Src kinase Yes and beta-catenin revealed lower phosphorylation in response to this SOS1 inhibitor. The MIA PaCa-2 line was compared to the KRAS G12C-mutated BH1362 NSCLC cell line in respect to SOS1 inhibition (Fig. 6C). Results show similarity to Aspc1 with decreased phosphorylation of CREB, EGFR and ERK1/2 and similar effects for GSK3beta, beta-catenin, Pyk-2 and Src kinases Src and Yes.

## Discussion

Pancreatic cancer makes up for 3% of all cancers in the Unites States (US) and accounts for 7% of all cancer deaths with increasing occurrence [4]. Median survival in response to treatment by chemotherapies is low and may not exceed 12–18 months [36]. Mutations of* KRAS* in PDACs are highly frequent, observable in approximately 70–95% of cases. These mutations result in a fixation of KRAS in the GTP-bound state and permanent activity, independently of extracellular stimuli, thus vigorously driving cancer growth [10, 37–40]. Suppression of mutated KRAS expression results in tumor regression of pancreatic tumors [41]. However, since G12D is the most common alteration of KRAS in PDAC these tumors are not amenable to treatment with G12C-specific agents, in contrast to many lung cancers and the 2% subset of pancreatic cancer patients carrying a G12C mutation that has been successfully hit. *KRAS* mutations are linked to more aggressive pancreatic cancers and a dismal prognosis [15, 42].

The advantage of inhibition of mutant KRAS is the avoidance of toxicity against normal tissues expressing wild-type KRAS. However, the RAF-MEK-ERK kinase cascade downstream of KRAS is indispensable for cells and recruitment of compensatory mechanisms cause resistance to monotherapy [43]. KRAS G12C is not stably locked in the GTP bound state and is still dependent on activation by upstream RTKs for GEF-mediated reloading [44, 45]. Inhibitors of the GEF SOS1 prevent the SOS1-KRAS-GDP interaction, thus impairing GTP loading and KRAS activity exchange [21]. The first SOS1 inhibitor in clinical trials, namely BI-1701963, was well tolerated and yielded stable disease in 7/31 KRAS mutated patients up to 18 weeks [46]. The search for inhibitors of SOS1 has yielded four compounds so far, namely BAY-293, MRTX0902, BI-3406 and BI-1701963, that inhibit KRAS-dependent tumor growth in vitro and in xenograft models [21, 25, 26]. The SOS1 inhibitor BAY-293 is not suitable for clinical application but has a high affinity and is a useful agent to study the function of SOS1 in models [47]. Inhibition of SOS1 allows to hit KRAS in an indirect manner and for KRAS mutations differently of G12C [48, 49]. Furthermore, in EGFR-mutated NSCLC spheroids with wildtype KRAS BAY-293 synergizes with EGFR-TKIs to block cellular growth [35]. Inhibition of the KRAS downstream signaling cascade has shown limited efficacy and high toxicity but may be combined with KRAS or SOS1 inhibitors at lower dosage [50, 51]. Inhibitors of SOS1 may be less sufficient to achieve clinical responses in tumor patients as single agents and, thus, combinations are required to maximize the efficacy and to limit the toxicity against normal tissues. Pathways that have shown special vulnerabilities against drugs in KRAS mutated cells versus wildtype cells may be of special interest for SOS1 combinations [34]. For the present study we employed the pancreatic cancer cell lines MIA PaCa-2, frequently used to study KRAS G12C biology, AsPC1 a KRAS G12D mutated cell line and the tumorigenic BxPC-3 cells that shows epithelial morphology and moderate differentiation, but is unique in lacking a KRAS mutation [33].

KRAS-mutated cancers are distinguished by alterations in metabolic pathways including elevated glycolysis and glutaminolysis as well as increased breakdown of fatty acids and nucleotides [34, 49, 52, 53]. In colorectal cancer (CRC), KRAS mutant tumor cells increased glycolysis and glutamine utilization resulting in cell death upon inhibition of glyceraldehyde 3-phosphate dehydrogenase (GAPDH) [54, 55]. In the tumor microenvironment (TME) increased activity of glucose transporter GLUT1 and hexokinase II (HK2) compensate for the irregular vascularization [41, 56]. The HK2 inhibitor 2-DG impairs cell proliferation and viability, especially in combination with BAY-293 for all 3 pancreatic cancer cell lines tested [57]. In particular, in NSCLC carrying mutant KRAS cells are sensitive to glucose deprivation or treatment with metformin, an inhibitor of mitochondrial complex I and of pancreatic cancer cell respiration [58]. Treatment with metformin in *KRAS* wildtype patient-derived xenograft (PDX) models had a minor antitumor effect but metformin treatment inhibited *KRAS* G12D tumor growth significantly [59]. This finding would be compatible with the unique effect of the AsPC1 cell line in contrast to the wildtype and G12C-mutated cell lines tested here.

Cancer cells show aerobic glycolysis, the well-known Warburg effect, and dichloroacetate (DCA) diverts the glucose utilization from aerobic glycolysis to respiration by inhibition of mitochondrial pyruvate dehydrogenase kinase 1 (PDK1) [60, 61]. DCA elevates the concentration of mitochondrial reactive oxygen species (ROS) and may lead to apoptotic cell death but exhibited no synergy with BAY-293 [61, 62]. Metformin has been reported to impair cell proliferation and survival preferentially in cells carrying KRAS mutations [63, 64]. In particular, in KRAS/LKB1 co-mutated tumors metformin synergizes with cisplatin [65]. Linsitinib is a dual inhibitor of the IGF-1 and insulin receptors (IR) that is critical for cell survival and shows marked synergy with BAY-293 [66, 67]. In conclusion, 2-DG, metformin and linsitinib modulate the cellular glucose metabolism and act synergistically with BAY-293.

Mutations in RAS cause permanent overaction of this G-protein and result in increased cell proliferation and survival due to boosted signaling via RAS-RAF-MEK-ERK and other pathways [68]. The first MEK1/2 inhibitor PD098059 was followed by trametinib (MEKinist™), as the first clinically useful MEK inhibitor applied as agent to treat BRAF-mutated melanomas [69–71]. Combined inhibition of mTOR by rapamycin and of MEK by trametinib achieved tumor suppression in lung cancer models carrying KRAS mutations [67]. Our results showed that both trametinib and PD098059 work synergistically with BAY-293 in case of BxPC3 and AsPC1 but not against MIA PaCa-2. Tumors with KRAS mutations proved more sensitive to CDK inhibitors compared to wild-type tumors [72]. Inhibition of both MEK and CDK4/6 by palbociclib induced responses in mutant KRAS colorectal and lung cancer models [73, 74]. The present tests revealed no activity of BAY-293—rapamycin combinations and palbociclib was antagonistic in case of AsPC1. The pan-CDK inhibitor flavopiridol was reported to exert marked toxicity in KRAS-mutated NSCLC cells [75]. Here, this compound synergized with BAY-293 for the KRAS-mutated cell lines but not for the wildtype BxPC3 cell line. Our combination experiments with BAY-293 and ERK/mTOR as well as CDK inhibitors confirm the results showing synergism of SOS1 inhibition and these modulators.

Other possible additions to wildtype KRAS inhibitors may comprise cytotoxic anticancer drugs. In wildtype EGFR NSCLCs, a platinum/pemetrexed regimen was inferior for KRAS-mutant versus wildtype KRAS patients and this type of chemoresistance may be due to mutated KRAS-dependent induction of NRF2-mediated cellular stress response [76, 77]. Here, cisplatin proved synergistic with BAY-293 in case of ASPC-1 and pemetrexed was active in combination, except for the antagonistic interaction in case of MIA PaCa-2. KRAS mutated NSCLC cells proved highly sensitive to treatment with TNF-related apoptosis-inducing ligand (TRAIL) and 5-fluorouracil (5-FU) [78]. Several of the chemotherapeutics were reported to kill selectively colon cancer *KRAS* mutant cells, in particular drugs targeting DNA topoisomerases, such as camptothecin derivatives and anthracyclines such as doxorubicin [79]. However, the synergistic interaction of BAY-293 and doxorubicin was limited to BxPC3 in our investigation. In contrast, SN-38 (7-Ethyl-10-hydroxycamptothecin) the active in vivo derivative of irinotecan, exhibited high synergistic activity with BAY-293 against MIA PaCa-2 and AsPC1 but not against wildtype BxPC3.

Inhibition of KRAS by the SOS1 inhibitor BAY-293 is expected to disturb the several downstream signaling pathways via Akt, the MAPK pathway or via RAL in dependence of the specific KRAS mutation and the cellular background. According to the present results, MIA PaCa-2 KRAS G12C is affected by BAY-293 via regulation of AKT, p70 S6 kinase and kinases such as Pyk-2 and Src kinases Src and Yes. In contrast, the effects of BAY-293 in Aspc1 cells carrying a G12D KRAS mutation result in altered phosphorylation of CREB, EGFR, ERK1/2 and Src kinases. The response to BAY-293 in BH1362 NSCLC KRAS G12C cells was similar to the alterations found in Aspc1 cells, namely concerning CREB, EGFR and ERK1/2 with additional changes in beta-catenin and Src kinases.

Akt controls cellular growth, proliferation, metabolism as well as survival and migration resulting in enhanced tumorigenesis and induction of chemoresistance in various tumor types [80–82]. Akt activates mTORC1, that in turn phosphorylates ribosomal protein S6 kinase (S6K) resulting in altered protein translation and SOS1 inactivation [83]. Phosphorylation of Thr389 correlates with p70 kinase activity in vivo and phosphorylation at Ser411, Thr421 and Ser424 activate p70 S6 kinase via reversal of pseudosubstrate suppression [84]. PYK2 (proline-rich tyrosine kinase 2) is a downstream mediator of the mutant KRAS signaling cascade and knockdown of PYK2 suppressed tumor growth in PDAC xenografts [85]. Furthermore, regulation of KRAS occurs by phosphorylation of tyrosine residues by members of the Src family of tyrosine kinases (SFKs), three of which, Src, Fyn, and Yes, are expressed in pancreatic cancer cell lines [86]. Src-mediated phosphorylation of KRAS on Tyr^32^ and Tyr^64^ alters the protein conformation that inhibit the function of KRAS [87].

Active CREB was found overexpressed in lung adenocarcinoma in dependence of mutant KRAS signaling [78]. Elevated expression of CREB was detected in K-RAS(V12)-transformed murine fibroblasts and K-RAS(V12)-mutated human tumor cells [88]. Silencing of CREB expression or application of the KG-501 inhibitor suppressed the malignant phenotype of K-RAS(V12) transformants. Accordingly, inhibition of CREB in KRAS-mutated PDACs sensitized the cancer cells to MEK- and AKT-directed therapy in [89].

## Conclusion

Inhibition of mutated KRAS has concentrated on covalent inactivation of Cys12 of KRAS G12C, on blocking KRAS downstream signaling, or prevention of KRAS–GEF interactions [90, 91]. In 2019 and 2020, Hillig et al. at Bayer [92] and Hofmann et al. at Boehringer Ingelheim [21] independently reported aminoquinazolines as disruptors of the KRAS SOS1 interaction. The aminoquinazoline scaffold is a moiety of EGFR inhibitors such as erlotinib, gefitinib and afatinib [93] but this reactivity has been eliminated for the SOS1 inhibitors. The BAY-293 compound is a potent blocker of the interaction between KRAS and SOS1 and binds to the same SOS1 pocket as other compounds developed [25, 92]. This inhibitor proved active in 2-dimensional cell culture (2-D) and reduced active KRAS-GTP levels in HeLa (RAS-WT, IC_50_ 410 nM) and Calu-1 cells (RAS-G12C, IC_50_ 200 nM). In a panel of 60 cancer cell lines BAY-293 displayed a relatively broad anticancer activity spectrum. Interestingly, all SOS1 inhibitors BAY-293, BI-3406 and RM-0331 target essentially the same binding pocket of this GEF, establishing BAY-293 as model compound for in vitro studies [25, 92]. BI-3406 requires tumor cells in 3D aggregates for activity, an arrangement that is characterized by increased MEK-ERK activity and higher dependency on KRAS activity [94]. SOS1 inhibitors effect a partial inhibition of KRAS signaling and obviously need to be combined with other drugs to yield marked anticancer effects and prevent drug resistance. Mutated KRAS reveals synthetic lethality with a range of compounds that may be useful in drug combinations [95]. In particular, KRAS-driven cancers show upregulated glycolysis, as well as altered glutamine utilization and mitochondrial function [96].

**Fig. 7:**
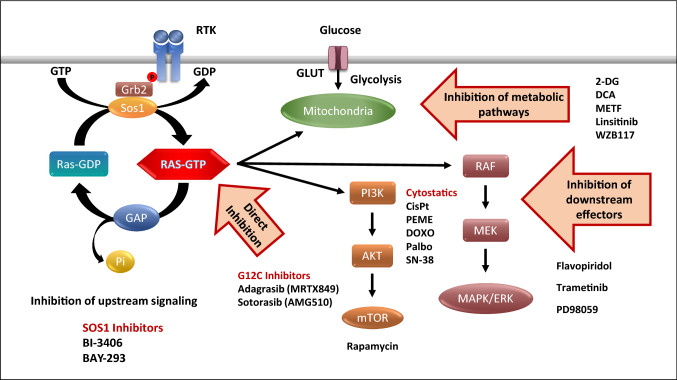
The GTPase KRAS is activated by Receptor tyrosine kinases like EGFR and stimulates proliferation after recruiting growth factor receptor bound protein 2 (Grb2) and of SOS1, a KRAS-guanine exchange factor (RAS-GEF). SOS1 inhibitors, KRAS G12C inhibitors and combinations with modulators of downstream signaling and of glucose utilization as well as various chemotherapeutics are suited to increase the impact of BAY-293

The present results demonstrate that special vulnerabilities of KRAS-triggered tumor cells can be exploited successfully in drug combinations targeting different pathways (Fig. 7). That SOS1 inhibitors can be combined with *KRAS* G12C inhibitors for the respective tumors and inhibition of downstream signaling is actively pursued in clinical studies. Interference with glucose uptake and metabolization at different levels seems to synergize with SOS1 inhibition as well as a range of conventional chemotherapeutics. The efficacy of all combinations is critically dependent on tumor type and on specific KRAS mutations. Furthermore, the distinct signaling pathways inhibited by BAY-293 must be taken into account for considering kinase inhibitors.

## Data Availability

Data may be available from the authors upon reasonable request.
